# Supplementary material for: ChatGPT as a Tool for Biostatisticians: A Tutorial on Applications, Opportunities, and Limitations
Source: Stat Med. 2025 Oct 23;44(23-24):e70263. doi: 10.1002/sim.70263 (PMC12548020; doi:10.1002/sim.70263)
Supplement: Supplementary file 1 — Data S1. Supporting Information. [file SIM-44-0-s001.zip › Supplementary Materials Additional.zip!/3.1_Systematic_Reviews_and_Meta_aspects/3.1_Data Extraction - Base Prompt 4o - Chat 6.pdf]

Use Case Meta-Analysis - Data Extraction - Base Prompt 4o - Chat 6

Rudloff et al. - 2009 - Concurrent lobular neoplasia increases the risk of ipsilateral breast cancer recurrence in situ treated with breast-conserving therapy.pdf
PDF

Vidali et al. - 2012 - Conservative treatment of breast ductal carcinoma in situ results of an Italian multi-institutional.pdf
PDF

The aim is to perform a meta-analysis on total ipsilateral local recurrence rates at 10 years in cases of ductal carcinoma in situ

Data inclusion rules are:
To ensure the validity of the follow-up data, the results should be as close to 10 years follow-up (minimum) as the information in each study allows. If there is more than one publication from an institution or group, the most recent study with appropriate follow-up will be used to extract 10-year data.

Please provide:

- the rate of ipsilateral local recurrence for conservative surgery with additional radiotherapy (no other interventions or drugs allowed) after 10 years
- the number of eligible patients
- period of data collection
- duration of follow-up
- type of study
- age of patients (mean or median, maximum and minimum)
- treatment modalities

(Please provide a dataframe with one entry for each study, and column names "study", "rate", "n_patients", "period", "follow_up", "study_type", "age", and "treatment". Do not provide additional text).

Here is the extracted data in the requested format:

```python
import pandas as pd

data = [
    {
        "study": "Rudloff et al. 2009",
        "rate": 0.119,
```

```
        "n_patients": 91,
        "period": "1991–1995",
        "follow_up": "11.8 years (median)",
        "study_type": "Retrospective cohort",
        "age": "Median 55 (range 26–89)",
        "treatment": "Conservative surgery + whole breast radiotherapy"
    },
    {
        "study": "Vidali et al. 2012",
        "rate": 0.096,
        "n_patients": 586,
        "period": "1985–2000",
        "follow_up": "11.3 years (median)",
        "study_type": "Retrospective multi-institutional cohort",
        "age": "Median 55 (range 29–84)",
        "treatment": "Conservative surgery + whole breast radiotherapy ± boost"
    }
]

df = pd.DataFrame(data)
df
```

Or visually:

study	rate	n_patients	period	follow_up	study_type	age	treatment
Rudloff et al. 2009	0.119	91	1991–1995	11.8 years (median)	Retrospective cohort	Median 55 (range 26–89)	Conservative surgery + whole breast radiotherapy
Vidali et al. 2012	0.096	586	1985–2000	11.3 years (median)	Retrospective multi-institutional cohort	Median 55 (range 29–84)	Conservative surgery + whole breast radiotherapy ± boost
